# FIN-EGFRprint: a Finnish real-word study on treatments and outcomes in advanced NSCLC with common EGFR mutations

**DOI:** 10.2340/1651-226X.2026.44731

**Published:** 2026-02-19

**Authors:** Aija Knuuttila, Lalli O Nurmi, Petri M. Vänni, Eija K. Heikkilä, Joanne Edwards, Monica H. Ekblom, Irene Luccarini, Maria Silvoniemi

**Affiliations:** a Department of Pulmonary Medicine, Heart and Lung Center, and Cancer Center, Helsinki University Central Hospital, Helsinki, Finland; b Nordic Healthcare Group Department, Espoo, Finland; c Johnson & Johnson; d Department of Pulmonary Medicine, Turku University Hospital, Turku, Finland

**Keywords:** carcinoma, non-small-cell lung, receptor, epidermal growth factor, protein kinase inhibitors, survival rate, treatment outcome, disease progression

## Abstract

**Background and purpose:**

The treatment of advanced non-small cell lung cancer (aNSCLC) with common epidermal growth factor receptor (cEGFR) mutations has evolved substantially over the last 15 years, with the discovery of activating epidermal growth factor receptor (EGFR) mutations and introduction of first-, second- and third generation (gen) EGFR tyrosine kinase inhibitors (TKIs) as first-line therapy. This retrospective observational study aimed to evaluate whether the introduction of these treatments has led to improved ‘real-world’ outcomes over time by analysing time to next treatment (TTNT) and overall survival (OS).

**Patient/material and methods:**

Patients (*n* = 379) with EGFR exon 19 deletion (Del19) or exon 21 L858R (L858R) substitution and aNSCLC were identified from two Finnish university hospital data lakes between 2010 and 2023. TTNT and OS were analysed from first-line treatment initiation using Kaplan–Meier survival and multivariable Cox regression analyses. Patients were stratified into three cohorts based on date of diagnosis and which TKIs were available at that time (1st-gen: 2010–2016, 2nd-gen: 2017–2020 and 3rd-gen: 2020–2023).

**Results:**

The use of chemotherapy as first-line therapy declined from 32% (2010–2016) to 6% (2020–2023), while 80% of patients received 3rd-gen TKIs as first-line treatment in 2020–2023. Median TTNT improved over time (9.7 to 13.2 to 21.6), with a significant improvement in 2020–2023 versus 2010–2016 (HR: 0.46; 95% CI: 0.33–0.64; *p* < 0.001). Median OS also increased over time (19.1 to 23.9 to 29.3 months) and was significantly higher in 2020–2023 versus 2010–2016 (HR: 0.56; 95% CI: 0.39–0.82; *p* = 0.002).

**Interpretation:**

‘Real-world’ treatment outcomes for aNSCLC with cEGFR mutations have improved over time likely due to the transition from 1st- to 3rd-gen TKIs. However, real-word survival with TKIs remains lower than clinical trials results emphasizing the unmet need.

## Introduction

Epidermal growth factor receptor (EGFR) mutations are identified in approximately 10–20% of Caucasian patients and 40–60% of Asian patients with non-squamous non-small-cell lung cancer (NSCLC) [1]. They are predominantly found in exons 18–21 of the EGFR gene, with the most common being in-frame deletions in exon 19 (Del19) and the Leu858Arg substitution (L858R) in exon 21. Together, these two alterations represent around 90% of all EGFR mutations and are referred to as common epidermal growth factor receptor mutations (cEGFR) [2]. These mutations are associated with marked sensitivity to tyrosine kinase inhibitors (TKIs), the introduction of which has significantly improved both prognosis and treatment strategies for patients with NSCLC. Finnish ‘real-world’ data are highly valuable for investigating outcomes in NSCLC with EGFR mutations, supported by a nationally standardized reimbursement timeline for EGFR TKI therapy, a centralized healthcare system, and high EGFR molecular testing rates. Moreover, electronic health records from multiple hospital systems are collectively accessible through data lake technology and can be combined with national data for reimbursed drugs, facilitating longitudinal research.

ESMO guidelines recommend that all patients with a sensitising EGFR mutation should receive first-line EGFR TKIs irrespective of clinical parameters [3]. Since the approval of the first EGFR-TKI in 2003, various generations of TKIs have emerged. Gefitinib and erlotinib are classified as 1st-gen TKIs, afatinib and dacomitinib as 2nd-gen TKIs, and osimertinib as a 3rd-gen TKI. In Finland, erlotinib (1st-gen) was granted reimbursement status in 2007, gefitinib (1st-gen) in 2010, afatinib (2nd-gen) in 2016, and first-line osimertinib (3rd-gen) in 2020.

TKIs became the standard of first-line care for NSCLC with EGFR mutations after clinical trials demonstrated the superior efficacy of gefitinib and erlotinib over platinum-based chemotherapy [4–7]. Subsequently, new generations of TKIs were developed due to concerns surrounding treatment resistance and tolerability. The LUX-Lung 7 trial showed that the 2nd-gen TKI afatinib improved progression-free survival (PFS) compared with gefitinib [8]. Moreover, the 3rd-gen TKI osimertinib demonstrated significantly longer PFS than platinum-based chemotherapy plus pemetrexed in patients with T790M-positive advanced NSCLC who had progressed on first-line gefitinib, erlotinib or afatinib [9]. Osimertinib was also shown to improve PFS and overall survival (OS) compared with erlotinib and gefitinib in a head-to-head clinical trial in untreated advanced non-small cell lung cancer (aNSCLC) patients with EGFR mutations [10, 11].

‘Real-world’ results on the impact of first-line osimertinib in advanced NSCLC with EGFR mutations are lacking from Finland. A ‘real-world’ retrospective study using the Finnish national Special Reimbursement Registry (*n* = 1498) showed that first-line afatinib was associated with improved survival compared to gefitinib, while osimertinib was not included as it was not reimbursed during the study period [12]. Another Finnish study reported survival outcomes in metastatic NSCLC treated with TKIs but did not compare different TKI generations [13]. Other Nordic and European ‘real-word’ studies have shown conflicting results. A nationwide Norwegian ‘real-world’ study showed improved OS in patients with advanced NSCLC with EGFR mutations (*n* = 449) when comparing the period of 2015–2019 with 2020–2022, suggesting that the improvement coincided with the availability of osimertinib in the latter period [14]. In contrast, real-world studies from the UK and USA reported no OS difference between first-line osimertinib and first-generation TKIs [15, 16].

The aim of this study was to evaluate whether ‘real-world’ treatment outcomes improved between 2010 and 2023, during which 1st-, 2nd- and 3rd-gen TKIs were sequentially introduced as first-line therapies for aNSCLC with cEGFR mutations. Time to next treatment (TTNT) and OS were assessed across the periods when the different generations of TKIs were predominantly used in Finland.

## Patients/material and methods

### Study design

This retrospective observational study utilised individual-level data from hospital data lakes and national registers in Finland. The study included first-line aNSCLC patients with cEGFR mutations, including del19 or L858R, from Helsinki University Hospital (HUS) and Turku University Hospital (TYKS), which together treat 41% of NSCLC patients in Finland. Patients were followed from baseline (up to 3 months prior to diagnosis) until death or the end of the study period.

### Data sources

The data lakes of HUS and TYKS were used to collect patients’ electronic health records. Information about TKI use was collected from the Medication Reimbursement Register of the Social Insurance Institution of Finland. Prior to analysis, data were linked using a unique individual identifier and pseudonymised by Findata, the data permit authority, in accordance with the Act on the Secondary Use of Health and Social Data (552/2019). All analyses were conducted within Findata’s secure Kapseli platform.

### Study population

This study included adult patients (≥18 years of age) who were diagnosed with aNSCLC (excluding ICD-10 code C34.X6) during 1.1.2010–30.9.2023 and had a cEGFR mutation. Patients were followed until the end of the study period, 31.12.2023. Advanced NSCLC patients were identified based on having received treatments for advanced disease, including: (1) chemotherapy treatment without surgery or radiotherapy within 2 months of this treatment, (2) 1st- or 2nd-gen TKI treatment with or without surgery or radiotherapy and (3) 3rd-gen TKI treatment except adjuvant treatment with surgery (given within 2 months after surgery) (Supplementary Figure 1).

### Time periods

Time periods for the study were defined based on the approval of reimbursement status for 1st-gen TKIs (erlotinib, November 2007; gefitinib, November 2010), a 2nd-gen TKI (afatinib, December 2016) and first-line 3rd-gen TKI (osimertinib, October 2020). Accordingly, three time periods were defined: 1 January 2010–31 December 2016; 1 January 2017–30 September 2020 and 1 October 2020–31 December 2023. The first period (1 January 2010–31 December 2016) was defined to begin when EGFR testing data became available, although erlotinib was reimbursed as early as 2007.

### Outcome measures and statistical analysis

Descriptive statistics are presented as means (standard deviations) for continuous variables and numbers (%) for categorical variables. The Kruskal–Wallis test (for continuous variables) and χ^2^ test for trend (for categorical variables) were used to assess changes in patient characteristics over time. Metastasis, stage and ECOG value records were extracted from physicians’ notes in the electronic health records using text mining and subsequently validated manually. TTNT was used as a proxy indicator for PFS. Both TTNT and OS were analysed using Kaplan–Meier (KM) estimates from the start of the first-line treatment. Group differences were assessed using multivariable Cox proportional hazards regression analysis adjusted for gender, age groups, Stage EGFR mutation type, ECOG value as well as CNS, liver or bone metastasis. Analyses were performed using R statistical software. To ensure patient anonymity, only results with data from at least five patients were reported.

## Results

### Patient characteristics

A total of 379 patients with aNSCLC with cEGFR mutations were identified among NSCLC patients diagnosed during the period 1.1.2010–30.9.2023. Baseline characteristics stratified by time periods are shown in Table 1. The mean age of the cohort was 69.5 years, and 54% had an EGFR Del19 mutation. Most patients (85%) were diagnosed at stage IV, and 42% had metastases in the brain, liver or bone (Table 1). Patients diagnosed during 2020–2023 were older at diagnosis, with 38% aged > 75 years, compared with 23% in 2010–2016 and 31% in 2017–2020 (*p* = 0.0386). The proportion of patients having stage IV disease at diagnosis was higher during 2017–2020 (90%) and 2020–2023 (89%) than during 2010–2016 (78%, *p* = 0.016). No significant changes over time were observed in the proportion of Del19 or L858R mutations nor in the frequency of brain, liver or bone metastases. The use of chemotherapy as first-line therapy declined from 32% (*n* = 41) in 2010–2016 to 6% (*n* = 7) in 2020–2023. Third-generation TKIs were used only during 2020–2023, with 80% (*n* = 99) of patients receiving them as first-line treatment during that period.

**Table 1:** Patient characteristics by time periods.

Characteristics	All patients *n* = 379	Time periods			*P*-value
		1.1.2010–31.12.2016 *n* = 129	1.1.2017–30.9.2020 *n* = 127	1.10.2020–30.9.2023 *n* = 123	
***Sex, n (%)***					
Female	260 (69%)	86 (67%)	87 (69%)	87 (71%)	0.785
Male	119 (31%)	43 (33%)	40 (31%)	36 (29%)	
***Mean age (SD)***	**69.5 (11)**	**67.4 (11)**	**70.4 (11)**	**70.6 (11)**	
***Age group, n (%)***					
**< 65 years**	**117 (31%)**	**46 (36%)**	**32 (25%)**	**39 (32%)**	**0.0386**
**65–75 years**	**145 (38%)**	**53 (41%)**	**55 (43%)**	**37 (30%)**	
**> 75-year**	**117 (31%)**	**30 (23%)**	**40 (31%)**	**47 (38%)**	
***Stage, n (%)***					
**Stage IIIb**	**55 (15%)**	**28 (22%)**	**13 (10%)**	**14 (11%)**	**0.016**
**Stage IV**	**324 (85%)**	**101 (78%)**	**114 (90%)**	**109 (89%)**	
***Histology, n (%)***					
Adenocarcinoma	342 (90%)	113 (88%)	115 (91%)	114 (93%)	0.392
Other	32 (10%)	16 (12%)	12 (9%)	9 (7%)	
***EGFR mutation type, n (%)***					
Del19	205 (54%)	76 (59%)	68 (54%)	61 (50%)	0.328
L858R	174 (46%)	53 (41%)	59 (46%)	62 (50%)	
***ECOG grade, n (%)***					
0	21 (6%)	< 5	#	9 (7%)	0.220
1	67 (18%)	20 (16%)	29 (23%)	18 (15%)	
≥ 2	15 (4%)	< 5	< 5	8 (7%)	
Missing	276 (73%)	103 (80%)	85 (67%)	88 (72%)	
***Smoking status, n (%)***					
Never	80 (21%)	25 (19%)	32 (25%)	23 (19%)	0.671
Current smoker	15 (4%)	< 5	< 5	7 (6%)	
Ex-smoker	25 (7%)	#	#	8 (7%)	
Missing	259 (68%)	91 (71%)	83 (65%)	85 (69%)	
***Metastasis location, n (%)***					
Brain	62 (16%)	14 (11%)	28 (22%)	20 (16%)	0.053
Liver	51 (13%)	12 (9%)	19 (15%)	20 (16%)	0.224
Bone	112 (30%)	37 (29%)	36 (28%)	39 (32%)	0.814
Brain, liver or bone (at least one of each)	158 (42%)	47 (36%)	59 (46%)	52 (42%)	0.263
***First-line treatments, n (%)***					
Chemotherapy	68 (18%)	41 (32%)	20 (16%)	7 (6%)	
1st gen TKI	159 (42%)	83 (64%)	61 (48%)	15 (12%)	
*-Gefitinib*	*–93 (25%)*	*–41 (32%)*	*–37 (29%)*	*–15 (%)*	
*-Erlotinib*	*66 (17%)*	*–42 (33%)*	*–24 (19%)*	*–0*	
2nd gen TKI (*Afatinib*)	53 (14%)	#* (~3%)	46 (36%)	< 5	
3rd gen TKI (*Osimertinib*)	99 (26%)	0	0	99 (80%)	

The table summarizes baseline patient characteristics by time period, bold text highlights statistically significant differences across periods. The Kruskal–Wallis test was used to assess differences in age across time periods, and the χ² test was used to evaluate trends in other categorical patient characteristics. If the patient number is less than five (< 5), it is displayed as ‘< 5’. If the patient number is hidden (#), it means that displaying it would allow the calculation of the exact patient number. This is done to preserve participant anonymity and comply with the Finish regulation.

### Treatment progression from first to second line

**Figure 1:**
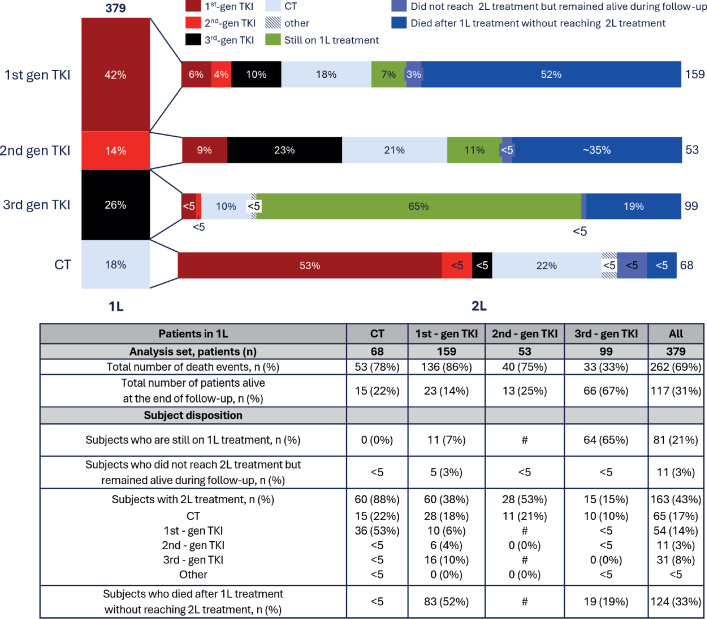
Treatment line progression from first to second line. The figure shows the proportion of patients treated with different generations of tyrosine kinase inhibitors (TKIs) or chemotherapy as first-line therapy, progression after first-line treatment, and the proportion remaining on first-line therapy during follow-up. If the patient number is less than five (< 5), it is displayed as ‘< 5’. If the patient number is hidden (#), it means that displaying it would allow the calculation of the exact patient number. This is done to preserve participant anonymity and comply with the Finish regulation.

The second-line treatments received by patients following first-line treatment are presented in Figure 1. Half of the patients (52%) receiving 1^st^-gen TKIs as first-line treatment died before reaching second-line. Among those who received second-line treatment, chemotherapy was the most common therapy (18%). A third (~35%) of the patients receiving 2^nd^-gen TKI as first-line treatment died before reaching second-line, their most common second-line treatment being a 3^rd^-gen TKI. Most patients (65%, *n* = 64) treated with 3^rd^-gen TKI in first-line were still on treatment at the end of the study period, given that osimertinib became available in Finland only a little over 3 years before the study period ended. Of those switching from 3^rd^-gen TKIs and receiving a subsequent therapy (*n* = 15), chemotherapy was the most-used option (*n* = 10, 67%). Most patients (88%) receiving chemotherapy in first-line received a second-line treatment (*n* = 60), predominantly TKIs (*n* = 43, 72%). Individual radiotherapies given in conjunction with TKI treatment were not evaluated in this study. Eighty-five per cent of patients were diagnosed with aNSCLC, while 15% were diagnosed with early-stage NSCLC (stages I–IIIa) before progressing to advanced-stage disease.

### Time to next treatment

**Figure 2:**
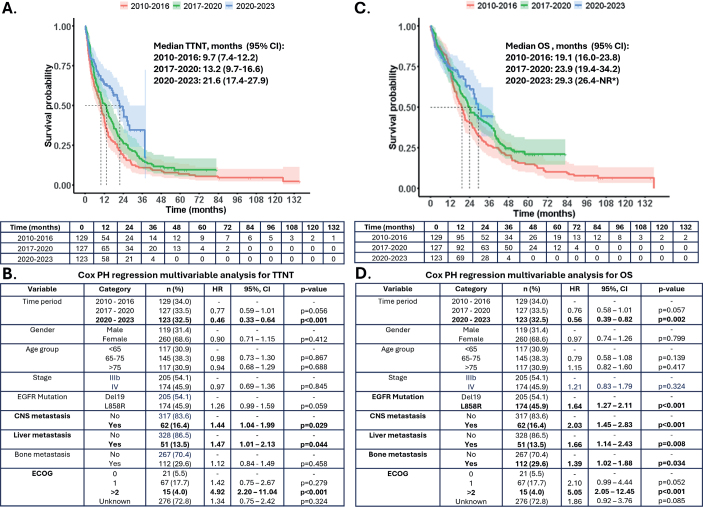
(A-B) TTNT and (C-D) OS by time periods. The figure illustrates Kaplan–Meier (KM) analyses of time to next treatment (TTNT) and overall survival (OS) across different time periods. Statistical differences between the time periods were assessed using adjusted multivariable Cox regression analysis. Time periods and patient-specific variables associated with improved or worsened TTNT or OS are highlighted in bold.

Median TTNT substantially improved over time: from 9.7 to 13.2 and further to 21.6 months over the three periods (Figure 2A). The TTNT increase from the first to the third period was statistically significant based on multivariable Cox proportional hazards analysis (HR: 0.46; 95% CI: 0.33–0.64; *p* < 0.001) The presence of CNS or Liver metastasis as well as ECOG performance status more than two were associated with shorter TTNT (Figure 2B).

### Overall survival

Median OS substantially improved over time: from 19.1 to 23.9 and further to 29.3 months over three periods (Figure 2C). The OS significantly increased from the first to the third period as shown with multivariable Cox proportional hazards analysis (HR: 0.56; 95% CI: 0.39–0.82; *p* = 0.002). Additionally, the L858R mutation, compared with the Del19 mutation, was associated with shorter OS. The presence of CNS, bone or liver metastases, as well as an ECOG performance status greater than two, were also associated with shorter OS (Figure 2D).

## Discussion and conclusion

In this retrospective observational study of aNSCLC with cEGFR mutations (2010–2023), we assessed changes in TTNT and OS across three time periods aligned with the introduction of 1st-, 2nd-, and 3rd-gen first-line TKIs. Median TTNT and OS improved from 2010–2016 to 2020–2023, likely due to advances in the treatment algorithm with the availability of 3rd-gen TKIs (80%) in the last period which were not available earlier [most frequently used (64%) treatment in the first time period was 1^st^ gen TKI]. Second generation was mainly used in the period 2017–2020 (36%), but TTNT and OS during that period were not significantly different from those beforehand and subsequently.

When comparing patient characteristics over time, patients diagnosed with aNSCLC with cEGFR mutations were significantly older in the latest time period, with 38% aged 75 years or older compared to 23% in the first time period. The proportion of patients diagnosed at stage IV was also higher in the latest time period than in the earliest one. This may also have been due to improvements in imaging techniques over time.

In our study, we did not directly compare treatment types but instead assessed their availability over time and associated outcomes. This approach minimised selection bias, as treatment practices have evolved over time and treatment choices were influenced by patient characteristics. The clinical variables that reflect these patient characteristics (such as ECOG performance status or variables related to patients’ general condition) are not widely available in ‘real-world’ data, impeding matching for treatment comparisons.

Our finding that OS improved with the transition from 1st-gen to 3rd-gen TKI treatment aligns with a meta-analysis of randomized controlled studies [17], although the OS in these ‘real-world’ patients was shorter than that observed with clinical trials. In a randomized trial studying patients with aNSCLC with cEGFR mutations (*n* = 556) by Ramalingam et al., superior OS was achieved with 3rd-gen TKIs than with 1st-gen TKIs (38.6 vs. 31.8 months) [10].

A Norwegian nationwide ‘real-world’ study compared OS in advanced NSCLC with EGFR mutations between 2015–2019 and 2020–2022 and, consistent with our findings, reported improved OS in the latter period coinciding with the availability of first-line 3rd-gen TKI treatment. However, the median OS in 2020–2022 (23.3 months) was shorter than in our study, and the analysis was not limited to cEGFR mutations as in our study [14]. Other European ‘real-world’ studies have shown varying results. A nationwide register study from Netherlands (*n* = 1109) showed no difference in OS between 3rd-gen and earlier TKIs in patients with cEGFR mutations (gefitinib:19.7, erlotinib: 23.2, afatinib:23.3 vs osimertinib: 22.8 months). However, the subgroup analysis showed that the OS benefit with osimertinib was restricted to patients with Del19 mutation [18]. A UK study (*n* = 336) showed no difference in OS between 3rd-gen and earlier TKIs (16.6 vs. 16.9 months) [15]. The UK study included uncommon EGFR mutations at a rate of 20%, which may explain the shorter OS compared to ours but also to the Netherland study which included only patients with cEGFR mutations. Nonetheless, these findings highlight the need for further studies investigating outcomes with 3rd-gen TKIs in larger cohorts and with longer follow-up and with separate consideration of EGFR mutational subtypes. The shorter OS observed in ‘real-world’ studies compared to clinical trials also underscores the need for further innovative treatments for aNSCLC.

Approximately half of the patients treated with first-line 1st-gen TKIs died before reaching second-line therapy, compared with fewer than 5% of those who received first-line chemotherapy. This may be explained by treatment practices in this earlier period, as discussed with local clinical experts, where chemotherapy was initiated while awaiting EGFR results, and TKI treatment for EGFR-mutation positive patients was started, before disease progression. This is supported by median TTNT being 4 months for first-line chemotherapy and 13 months for 1st-gen TKIs. In addition, among those receiving first-line chemotherapy, 72% received TKIs as second-line treatment. According to local clinical experts, chemotherapy was also given more often to patients with a better general condition [19] which may have further contributed to the lower mortality for first-line chemotherapy than for first-line 1st-gen TKIs.

Our study has some limitations. Its retrospective nature introduces potential confounding factors compared with prospective randomised trials. Moreover, follow-up for patients on 3rd-gen TKIs was limited, as first-line reimbursement in Finland began only in October 2020, leaving many still under treatment at the end of the follow-up period. Additionally, PFS was not available in the ‘real-world’ data (due to inaccessibility of imaging data), but TTNT was used as a proxy for PFS. However, TTNT and subsequent treatment availability may be affected by local practice patterns, reimbursement policies, toxicity-related changes, and physician preferences. In addition, in NSCLC with EGFR mutations, treatment may be continued beyond progression when subsequent therapies are not available. The information on histological type was obtained from ICD codes. Any further histological confirmation made by physicians is typically recorded in doctors’ notes and was not captured in this study.

Our study demonstrates that investing in treatment innovations has resulted in improved ‘real-world’ outcomes: treatment outcomes for cEGFR-positive aNSCLC patients improved from 2010 to 2023, coinciding with the treatment evolution over time and the introduction of 3rd-gen TKIs for first-line treatment in the most recent period (2020–2023). While treatment outcomes for aNSCLC patients have improved, ‘real-world’ survival remains poor and lower than clinical trial results, emphasising the existing unmet need in this patient population. In addition, as the proportion of older patients has increased, there is a growing need for well-tolerated and easily administered drugs, particularly when treatment durations are long.

## Supplementary Material



## Data Availability

Raw data are available upon research permit and data request from national health data access body. The data sharing policy of Johnson & Johnson is available at https://innovativemedicine.jnj.com/our-innovation/clinical-trials/transparency. Analysis of the data for the current study were made available by Company NHG and used under license for the current study and are not publicly available. Other researchers should contact NHG.
