# Supplementary figures and images for: FIN-EGFRprint: a Finnish real-word study on treatments and outcomes in advanced NSCLC with common EGFR mutations
Source: Acta Oncol. 2026 Feb 19;65:44731. doi: 10.2340/1651-226X.2026.44731 (PMC12927441; doi:10.2340/1651-226X.2026.44731)

Supplementary Figure 1: Flowchart for patient selection for the study

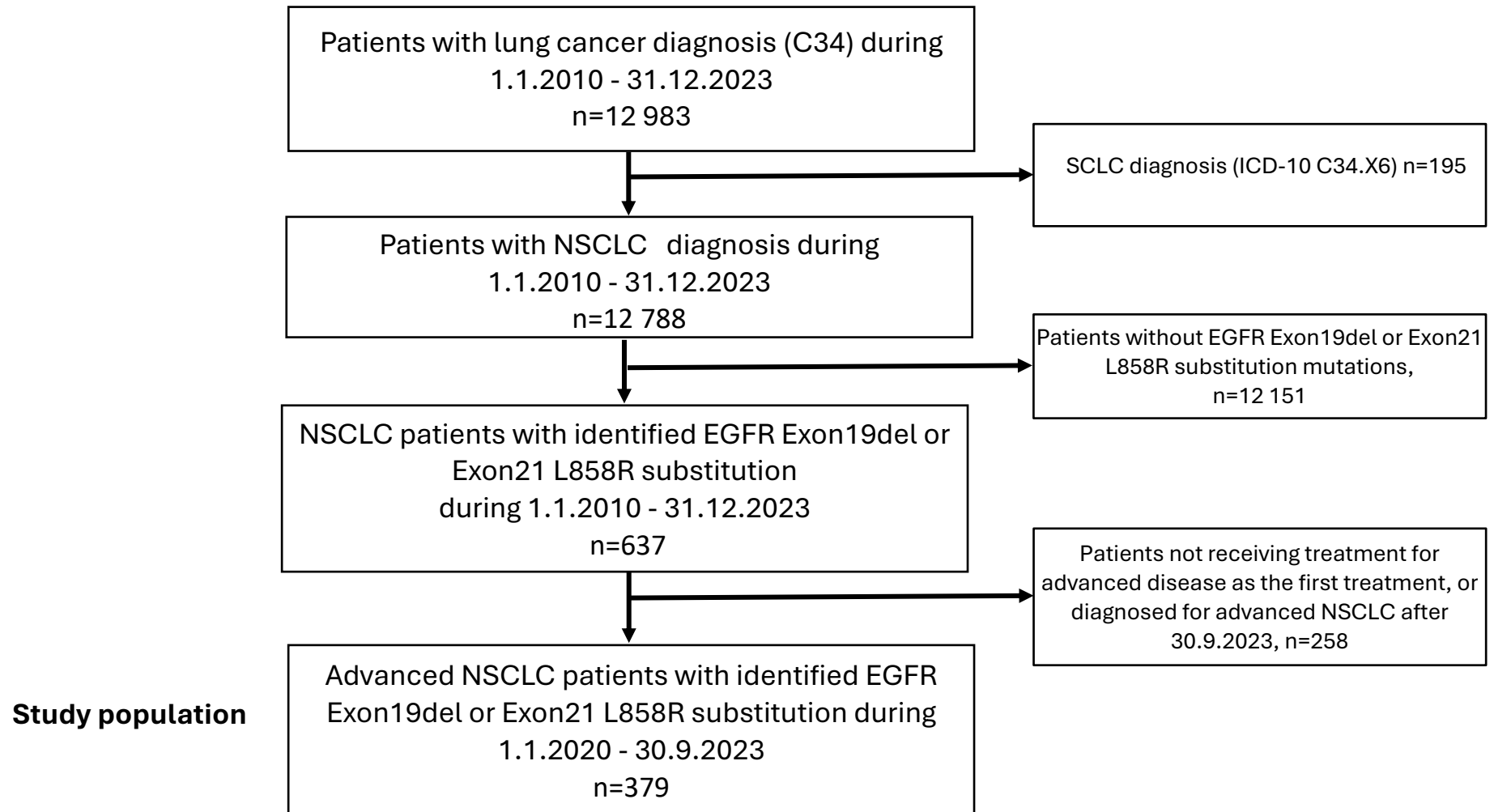

Supplement: Supplementary file 1 [file AO-65-44731-s1.pdf]
